# Alterations of upper-extremity functional muscle networks in chronic stroke survivors

**DOI:** 10.1007/s00221-024-06973-x

**Published:** 2024-12-23

**Authors:** David O’Reilly, Ioannis Delis

**Affiliations:** School of Biomedical sciences, University of Leeds, Leeds, UK

**Keywords:** Muscle networks, Motor control, Stroke, Muscle synergies, Clinical motion analysis, Redundancy, Synergy

## Abstract

Current clinical assessment tools don’t fully capture the genuine neural deficits experienced by chronic stroke survivors and, consequently, they don’t fully explain motor function throughout everyday life. Towards addressing this problem, here we aimed to characterise post-stroke alterations in upper-limb control from a novel perspective to the muscle synergy by applying, for the first time, a computational approach that quantifies diverse types of functional muscle interactions (i.e. functionally-similar (redundant), -complementary (synergistic) and -independent (unique)). From single-trials of a simple forward pointing movement, we extracted networks of functionally diverse muscle interactions from chronic stroke survivors and unimpaired controls, identifying shared and group-specific modules across each interaction type (i.e. redundant, synergistic and unique). Reconciling previous studies, we found evidence for both the concurrent preservation of healthy functional modules post-stroke and muscle network structure alterations underpinned by systemic muscle interaction re-weighting and functional reorganisation across all interaction types. Cluster analysis of stroke survivors revealed two distinct patient subgroups from each interaction type that all distinguished less impaired individuals who were able to adopt novel motor patterns different to unimpaired controls from more severely impaired individuals who did not. Our work here provides a nuanced account of post-stroke functional impairment and, in doing so, paves new avenues towards progressing the clinical use case of muscle synergy analysis.

## Introduction

A cerebrovascular event, commonly referred to as a stroke, causes long-term debilitating impairments to motor function (Crichton et al. 2016). Hemiparesis, muscle weakness, dyscoordination and fatigue are frequently observed among survivors who report difficulties carrying out activities-of-daily-living (Patel et al. 2002), in particular, those involving the upper-limb (Faria-Fortini et al. 2011). As patients reach chronic recovery stages, they learn to cope with their acquired motor deficits by adopting compensatory motor strategies such as recruiting extra degrees-of-freedom to perform a task compared to healthy individuals (e.g. greater trunk involvement during reaching (Cirstea and Levin 2000). The flexor and extensor synergies of the upper-limb are frequently observed abnormal motor patterns, the former involving increased shoulder abduction, elbow flexion and supination and wrist/finger flexion and the latter comprised of opposing joint motions (Brunnstrom 1970; Dipietro et al. 2007). However, these acquired patterns may or may not be adaptive towards overall motor function and consequently may represent a potential barrier to functional recovery that need careful monitoring from the outset of recovery (Jones 2017). Moreover, when impairment is assessed using qualitative assessments (e.g. Fugl-Meyer assessment (FMA) (Gladstone et al. 2002)), established compensatory strategies may also mask the actual neurological deficits of patients, making it difficult for clinicians to identify specific impairments and prescribe targeted interventions. Thus, current measures don’t fully characterise the complex interactions underpinning abnormal motor strategies, as even stereotypical strategies such as the flexor/extensor synergy have demonstrated subtle and contrasting multi-joint interactions (McPherson and Dewald 2019). Indeed, it has been recently highlighted that no single measure of motor impairment can effectively quantify activity and participation level outcomes among chronic stroke survivors (Bushnell et al. 2015). Thus, a current research gap resides in effective approaches to quantifying chronic stroke motor impairment that address these shortcomings in clinical evaluations.

Electromyography (EMG) technology has advanced significantly in recent years (Farina and Enoka 2023; McManus et al. 2020), developing into a genuine neural interface with the ability to enable clinicians to conveniently peer into the inner-workings of the human nervous system. Indeed, a prominent line of research exploiting this capacity is muscle synergy analysis, a neuroscientific approach that quantifies patterns of coordinated muscle activity (‘*muscle synergies*’) (Bruton and O’Dwyer 2018; Cheung and Seki 2021). In doing so, the extracted modules are analysed as the outputs of corticospinal neural circuitry, reflecting their functional organisation as they orchestrate task-specific movements (d’Avella & Bizzi 2005). Hence, this holistic approach has been of particular interest in clinical and rehabilitation research, as it may address shortcomings in the clinical evaluation of chronic stroke (Funato et al. 2022; Hong et al. 2021). Thus far, this approach has demonstrated great promise, characterising stereotypical motor patterns of stroke with direct links to patient impairments (Cirstea and Levin 2000; Clark et al. 2010; Roh et al. 2013). Recent developments by our research group have addressed important limitations in this approach that has hampered its progression as a clinical tool (O’Reilly et al. 2023; O’Reilly & Delis 2024; Ó’Reilly and Delis 2022), including restrictive model assumptions, the generalisability of extracted components, and both their functional and physiological relevance and interpretability (Alessandro et al. 2013). Our framework leverages network – and information – theoretic tools along with machine-learning (referred to as the network-information framework (NIF) to enable the novel capability of mapping networks of muscles to their functional consequences and, in doing so, characterising the diverse ways they cooperate to achieve task-goals (i.e. functionally -similar (redundant), -complementary (synergistic), -independent (unique). This innovation adds crucial nuance to the synergy concept that describes muscles as *‘working together’* towards task performance, showing they work together in functionally diverse ways concomitantly. Hence, this novel approach holds great potential to effectively address this current research gap and contribute towards a recent trend in network-theoretic approaches to temporal or frequency domain analyses of EMG signals aimed at progressing our clinical understanding of human movement (Houston et al. 2021; O’Reilly & Delis 2024; Roeder et al. 2024; Zhao et al. 2024).

In the current study, we aim to apply this established approach for the first time to chronic stroke survivors and, in doing so, characterise their motor impairments through a novel perspective to the muscle synergy. Towards this aim, taking single-trials of a forward pointing movement from 20 impaired and 17 unimpaired adults, we mapped pairwise muscle covariations to the trajectory of specific kinematic markers placed on the affected upper-limb and characterised their functionally-similar (redundant), -complementary (synergistic), and -independent (unique) contributions. We identified important structural similarities and differences across each muscle interaction type that not only aligned with findings from current muscle synergy analysis but also added substantially to our understanding of functional impairment post-stroke, reconciling important discrepancies in previous studies. Further, based on these structural alterations, we also stratified patient into two distinct subgroups in each interaction type that were all associated with functional and/or cognitive impairment and distinguished less impaired patients who adopted novel motor patterns from more severely impaired patients who did not. This study provides a nuanced account of functional impairment in chronic stroke, making a significant contribution towards the improvement of clinical assessment tools.

## Materials and methods

### Dataset and experimental setup

We conducted a secondary analysis on an opensource dataset (‘*MHH*’ (Averta et al. 2021), consisting of 20 chronic stroke survivors (Age: 49.88 ± 16.92, Chronicity: 13.37 ± 14.12 years (range: 2–56 years), 6 females, upper extremity FMA (FMA-UE) = 17.75 ± 2.05, Mini mental state examination (MMSE) = 27.9 ± 1.45) and 17 unimpaired controls (Age: ~46.8 ± 15.3years) where participants were asked to perform in random order a list of daily-living activities. Of these activities, we randomly selected 1 of the 3 trials performed of the forward pointing movement task (i.e. Task No. 9 of the SoftPro Protocol (Averta et al. 2021) for further analysis. This task required participants, who were sat at a table with their reaching arm laying pronated on the table, to point with their index finger and arm outstretched at a fixed-point straight ahead and then return to this starting position (Fig. 1.1(A) (Averta et al. 2019). The data captured from these trials included 12 single-channel EMGs (sampling rate = 2000 Hz) (Deltoideus pars clavicularis (**DC**), Biceps brachii (**BB**), Triceps brachii (**TB**), Flexor digitorum superficialis (**FDS**), Extensor digitorum (**ED**), Brachioradialis (**BR**), Flexor carpi ulnaris (**FCU**), Extensor carpi ulnaris (**ECU**), Pronator teres (**PT**), Flexor carpi radialis (**FCR**), Abductor pollicis brevis (**APB**), Abductor digiti minimi (**ADM**) (Fig. 1.2(A). Further, for brevity, 6 kinematics were curated from the 21 markers available (see original kinematic setup for MHH in (Averta et al. 2021) (sampling rate = 200 Hz) by combining the XYZ trajectories for each marker (i.e., multiplying X, Y and Z coordinates) and then determining the midpoint between redundant markers, thus providing 3D coordinates for the wrist (**WR**), forearm (**RU**), elbow (**ELB**), upper-arm (**H**), shoulder (**Sh**) and chest (**CS**) (Fig. 1.2(B). The EMGs were rectified and then low-pass filtered with a 4th order, zero-phase, Butterworth filter at 20 Hz while the kinematics did not undergo further pre-processing.

**Fig. 1:**
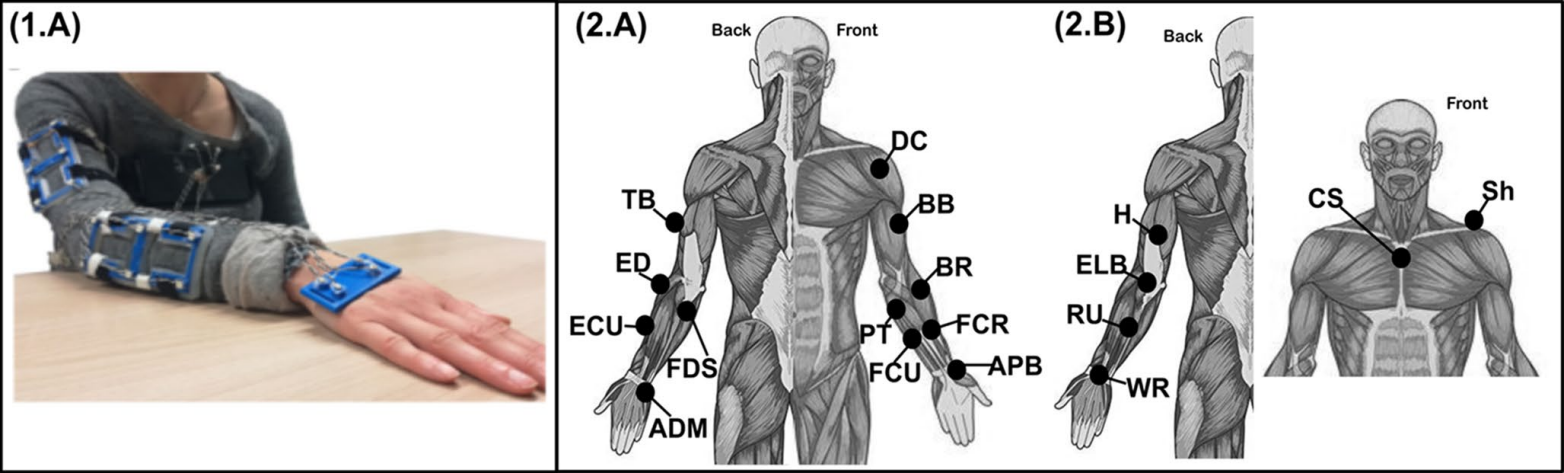
The initial and end position for the forward pointing task analysed in the current study (Averta et al. 2019, 2021). Starting with their arm laying pronated on a table, participants had to point forward with their index finger and arm outstretched at towards fixed-point straight ahead. The arm was then returned to the initial position at rest on the Table (**2.A**) The experimental setup for EMG sensors applied to 12 muscles of the affected side of 20 post-stroke participants and to 17 unimpaired controls. The muscles included were: Deltoideus pars clavicularis (DC), Biceps brachii (BB), Triceps brachii (TB), Flexor digitorum superficialis (FDS), Extensor digitorum (ED), Brachioradialis (BR), Flexor carpi ulnaris (FCU), Extensor carpi ulnaris (ECU), Pronator teres (PT), Flexor carpi radialis (FCR), Abductor pollicis brevis (APB), Abductor digiti minimi (ADM). (**2.B**) 6 kinematic marker coordinates in combined 3D space were analysed in this study and included: the wrist (WR), forearm (RU), elbow (ELB), upper-arm (H), shoulder (Sh) and chest (CS)

### Extracting networks of functionally diverse types of muscle interaction

We employed a computational approach we recently developed (i.e. the NIF) that decomposes the task-relevant information (i.e. task-specific muscle covariations) present among muscle pairings (e.g. [$\:{m}_{x}$,$\:{m}_{y}$]) and characterises their functionally -similar (i.e. redundant), -complementary (i.e. synergistic) and -independent (i.e. unique) contributions to task performance (𝜏) (O’Reilly et al. 2023; Ó’Reilly and Delis 2022) (Fig. 2.1(A). More specifically, the NIF begins with the principled dissection of task-relevant information from muscle pairings and individual kinematic trajectories (i.e. muscle covariations that predict task performance) using a partial information decomposition (PID) approach (Ince 2017; R. A. A. Ince et al. 2017). PID quantifies four distinct informational components that represent the different ways $\:{m}_{x}$ and $\:{m}_{y}$ provide information about 𝜏 including: redundancy (i.e. the task information shared equivalently in both $\:{m}_{x}$ and $\:{m}_{y}$ ($\:(\varvec{R}$) pink intersection), synergy (i.e. the task information found exclusively through the combination of $\:{m}_{x}$ and $\:{m}_{y}$ (($\:\varvec{S}$) orange shading) and two unique information quantities (i.e. the task information provided by $\:{m}_{x}$ that is not found in $\:{m}_{y}$ (($\:{\varvec{U}}_{1}$) magenta intersection) and vice versa (($\:{\varvec{U}}_{2}$) cyan intersection) (Fig. 2.1(B). These four quantities sum together to the joint mutual information between [$\:{m}_{x}$,$\:{m}_{y}$] and 𝜏 (i.e. the total task-relevant information found in [$\:{m}_{x}$,$\:{m}_{y}$] ($\:I({m}_{x},{m}_{y};{\tau\:})$) (Fig. 2.1(B).

**Fig. 2:**
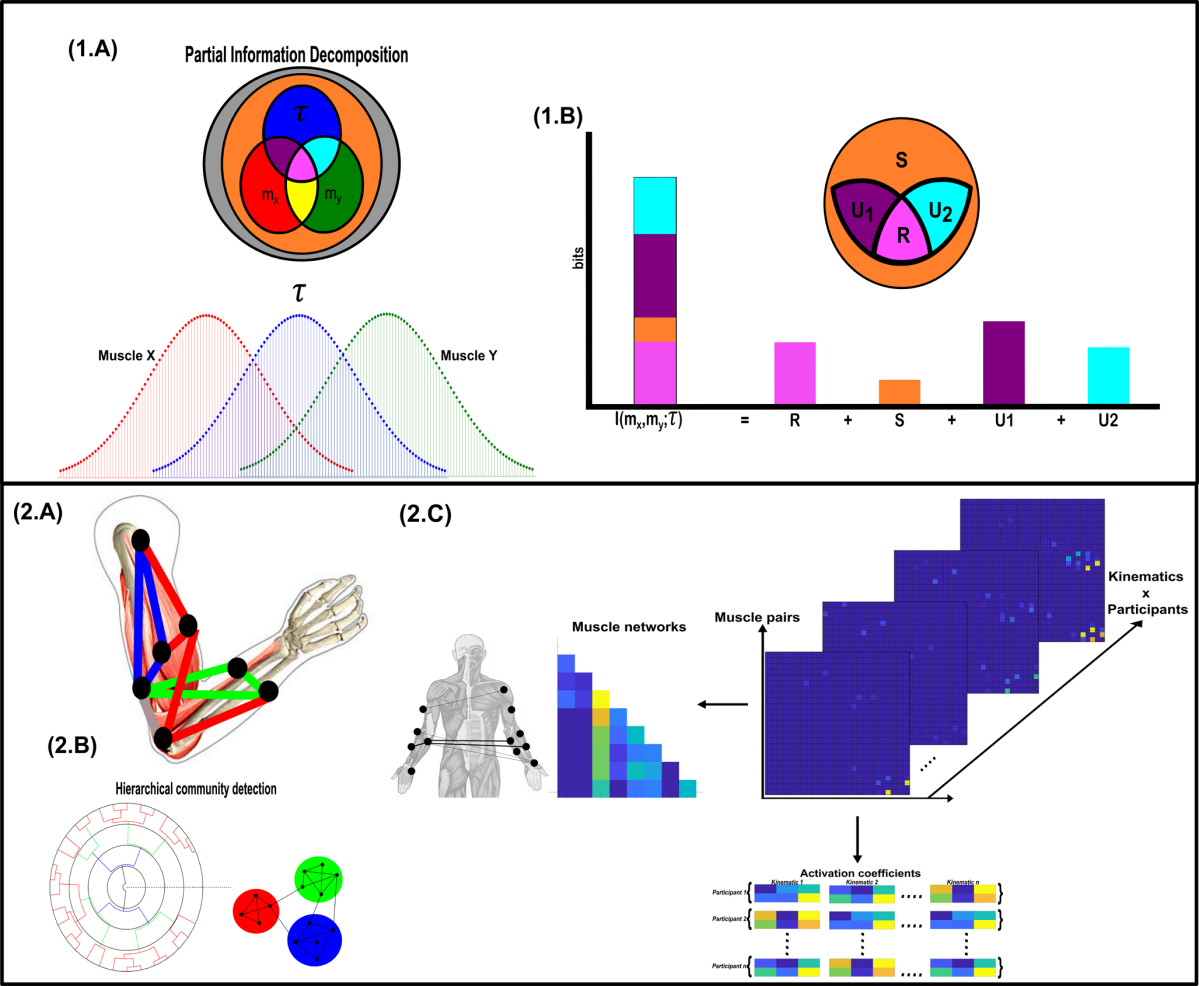
(**1.A**) Our established framework decomposes the shared information between pairs of muscles ($\:{m}_{x},{m}_{y}$) and a given task parameter (𝜏) using Partial information decomposition (PID). **(1.B)** This computation produces four distinct quantities: redundancy (i.e. task information common to both muscles (pink intersection)), synergy (i.e. task information exclusively revealed by observing both muscles together (orange shading)) and two unique information values for each muscle (i.e. task information that is present in $\:{m}_{x}$ that is not found in $\:{m}_{y}$ (magenta intersection) and vice versa (cyan intersection)). (**2.A**) Applying this computation to all unique muscle pairs produces networks of functionally diverse muscle interactions comprised of overlapping modular components (i.e. the yellow, green and grey connections representing interconnected modules). (**2.B**) To unravel this modular within the networks for each interaction type, we apply a hierarchical community detection protocol that identifies the optimal number of components to extract. (**2.C**) We apply projective non-negative matrix factorisation to the sparsified networks in vectorised form consisting of a specific type of interaction (i.e. redundant, synergistic or unique) and use the optimal number of components identified as an empirically derived input parameter

Applying this approach to all unique muscle pairings generates networks of muscle interactions for each type of functional contribution (i.e. redundant, synergistic and unique), kinematic marker and participant. For each network generated, we then identified statistically significant muscle interactions at the network level by applying a modified percolation analysis (Gallos et al. 2012), setting functional connections below this critical threshold to zero. Subsequently, we concatenated the sparsified networks across kinematics and participants, creating multiplex networks for each interaction type and experimental group. Overlapping muscle clusters (e.g. edge colours in Fig. 2.2(A) represent different functional muscle groups and illustrate muscles affiliated with more than one group) were then identified for each layer in the multiplex networks using a link-based community detection protocol that assigns each network connection to a cluster with the aim of maximising the number of possible links within each cluster (Fig. 2.2(B) (Ahn et al. 2010). A consensus partition was found across kinematics and participants by aggregating the clusterings from each layer into a single adjacency matrix and applying a conventional network community detection approach based on the Louvain algorithm (Blondel et al. 2008; Rubinov and Sporns 2010). Finally, the number of clusters identified then served as the input parameter into dimensionality reduction, where we employed a projective non-negative matrix factorisation (PNMF) method (Fig. 2.2(C) (Yang and Oja 2010). The input matrix for this algorithm includes the sparsified networks from a single interaction type (i.e. redundant, synergistic or unique) for all kinematics and participants of an experimental group in their vectorised form. The application of this framework here resulted in the extraction of co-occurring muscle network weightings and their corresponding kinematic- and participant-specific activation coefficients (Fig. 2.2(C). The following points summarise the steps involved in the NIF:

1. Apply PID to all unique muscle pairings and individual kinematic trajectories, generating networks of muscle interactions with diverse functional consequences (i.e. functionally-similar (redundant), -complementary (synergistic) and -independent (unique) (Fig. 2.1(A-B)).
2. Empirically sparsify each network, leaving functional connections that are considered significant with respect to the network’s percolation threshold.
3. Apply network community detection methods to the sparsified networks to determine the optimal number of components to extract using dimensionality reduction (Fig. 2.2(A)).
4. Extract muscle network components and their corresponding activation coefficients across participants and kinematic markers using PNMF (Fig. 2.2(B)).

Matlab code for the presented methodology is available here: https://github.com/DelisLab/Muscle_PID.

### Characterising post-stroke alterations in muscle network structure

To quantify the structural similarity of extracted muscle networks between experimental groups, we conducted a representational similarity analysis (RSA) whereby we quantified the 1-r distance between each impaired and unimpaired component of the same interaction type using Pearson’s correlation (Kriegeskorte et al. 2008). This procedure constructs a representational dissimilarity matrix (RDM) where lower values suggest a closer structural correspondence between a given pair of experimental groups’ extracted muscle networks. In doing so, this RDM will provide insight into the muscle networks that are shared or not between the experimental groups.

To determine subgroups of chronic stroke survivors based on the alterations in their muscle network structure, we employed RSA as described above except instead here, applied to the unsparsified versions of the corresponding kinematic-specific networks of all unimpaired and impaired participant pairings (i.e. individual unimpaired participant vs. individual impaired participant for the same kinematic), resulting in a set of RDMs ($\:C$) of dimension [No. of unimpaired participants ($\:{p}_{unim}$) x No. of impaired participants ($\:{p}_{im}$) x No. of kinematics ($\:k$)]. This matrix thus contains the distances between each pair of impaired and unimpaired participant in terms of the structure of their network interactions with respect to each kinematic marker. Subsequently, we computed the outer product of each RDM, creating a one-mode projection ($\:C$) with $\:k$ layers where each layer has dimension [$\:{p}_{im}$ x $\:{p}_{im}$] (Kerkman et al. 2020; Murphy et al. 2018). Each layer of $\:C$ thus contained the dot product similarity of the distances of each pair of impaired participants from each individual unimpaired participant for a given kinematic parameter. Nonsignificant distances were then empirically sparsified from each layer of $\:C$ using the modified percolation analysis (Gallos et al. 2012), and then the concatenated layers of $\:C$ were input into an agglomerative clustering algorithm with a complete linkage method to identify clusters of chronic stroke survivors. ~5 clusters of stroke survivors have been identified in previous established work (Scano et al. 2017). To more broadly define functional commonalities in the post-stroke population, we considered the first split in the dendrogram from the root node as an empirical bipartition the salience of which we determined in subsequent statistical analyses (see *‘Statistical analyses’* section of Materials and Methods section).

To characterise the alterations of functional muscle interactions following a stroke at a more local level (i.e. among specific submodules), we applied the network-based statistic (NBS) with threshold-free cluster enhancement (TFCE) to compare the muscle networks of specific interaction types (i.e. redundant, synergistic and unique) between experimental groups across kinematics (Smith and Nichols 2009; Zalesky et al. 2010). NBS follows the same principles underlying statistical parametric mapping for time-series data (Friston et al. 1994), whereby mass-univariate statistics are computed, in this case t-test statistics between corresponding network edges of both experimental groups across kinematics, while principally controlling for family-wise error. Thus, we can characterise network edge-level differences that are consistently present between impaired and unimpaired groups. Moreover, the incorporation of TFCE enables the enhancement of co-occurring topological differences among neighbouring network edges such that salient clusters of discriminative muscular dependencies can be identified (Baggio et al. 2018). This protocol was employed using opensource Matlab code: https://github.com/SNeuroble/NBS_benchmarking.

### Statistical analyses

Data pre-processing and framework application was conducted in Matlab 2023a software. To characterise the submodular structure of each extracted muscle network, we applied the Louvain algorithm to partition each muscle in the network into separate submodules (Blondel et al. 2008; Rubinov and Sporns 2010). These submodules are illustrated in the outputs as distinct colours on the corresponding nodes of a network overlayed on a human body model.

To determine if the stroke survivor subgroupings identified using RSA were associated with established measures of cognitive and motor impairment (i.e. MMSE and FMA-UE respectively), we used independent samples t-tests in SPSS Statistics 28. Significance was set a priori to *p* < 0.05.

## Results

### Functionally similar muscle networks

For both the healthy controls and post-stroke groups, we identified 5 clusters of functionally-similar muscle interactions across participants (R1-R5 (Fig. 3.2(A) = Unimpaired, Fig. 3.2(A) = Impaired). The strength of the most significant network edges (indicated by edge-width) and submodular structure (indicated here by node colour (Blondel et al. 2008; Rubinov and Sporns 2010) of each muscle network are presented above on human body models. Below, the corresponding kinematic-specific activation coefficients averaged across participants are depicted where node size indicates the magnitude of activation (Fig. 3.1–2(B). The RDM for all muscle network pairings between groups revealed structural similarities between the unimpaired R1-R3 and R5 with impaired R1, R4, R3 and R5 respectively (black framed squares (Fig. 3.3(A)). The R1 pairing between groups demonstrated the greatest similarity (1-*r* = 0.01) and involved prominent interactions between the FDS and both PT and EDU. The accompanying activation coefficients were also closely matched, although the activation for the post-stroke were less distinguished for the elbow and chest kinematics, presenting with a more uniform activation across kinematics (Fig. 3.1–2(B). The unimpaired R2 (comprised of strong connectivity between FCU-APB, FCR-PT and ADM-APB) was structurally consistent with the stroke groups’ R4 (1-*r* = 0.185 (Fig. 3.3(A)). A less distinguished scaling for specific kinematics was again found for the impaired R4 component (Fig. 3.1–2(B). Moreover, unimpaired R5 (redundant functional couplings between ECU and both APB and PT) was mostly preserved in R5 post-stroke (1-*r* = 0.07 (Fig. 2.3(A). Their kinematic activation differed with post-stroke participants involving the shoulder proportionally more compared to the upper-arm of the unimpaired controls (Fig. 3.1–2(B). Finally, impaired R3 mapped onto unimpaired R4 (1-*r* = 0.14 (Fig. 3.3(A)) but also to a much lesser extent with the unimpaired R3 (1-*r* = 0.81 (Fig. 3.3(A)). Indeed, both unimpaired R4 and impaired R3 comprised of significant redundancy between the ED and the FCU and to a lesser extent the BR. Meanwhile, the correspondence between R3 of both groups was found specifically among the FCR-ADM and FCR-APB couplings which were present to a relatively lower extent among the post-stroke samples’ R3. Impaired R2 did not correspond with any specific muscle network from the controls and comprised of prominent couplings between FDS and both ADM and APB, a set of functionally similar interactions that did not feature in any of the control groups’ networks.

Directly comparing the structure of participant- and kinematic-specific functionally similar muscles networks (i.e. prior to dimensionality reduction) between groups using RSA and then clustering the output across post-stroke participants into a global level bi-partition revealed a 13 member cluster (red cluster) and the remainder comprising the green cluster (Fig. 3.3(B)). We found that the red cluster had significantly lower MMSE scores (27.39 ± 1.45 vs. 28.86 ± 1.07 (t= -2.59, *p* < 0.05)) and non-significantly lower FMA-UE scores (17.62 ± 2.1 vs. 18 ± 2.1 (t= -0.39, (*p* > 0.05)) compared to the green cluster members. These functional differences were underscored by a greater aggregate distance of the less impaired green cluster patients from unimpaired controls across kinematics (illustrated by the aggregate RDM (Fig. 3.3(B)). This finding suggests the less cognitively impaired subgroup (green cluster) were more able to adopt novel motor patterns of functional similar muscle interactions (captured here as a greater representational distance) to effectively perform the pointing movement, thus distinguishing them from the red cluster participants.

Although the global structure of the functionally similar muscle networks was mostly consistent across groups, their submodular structure, quantified via their assortative mixing (i.e., the tendency of muscles to group with others with similar functional connectivity) (Blondel et al. 2008; Rubinov and Sporns 2010), provided further insight into differences in their functional interrelationships. Briefly, three submodules of similarly coupled muscles were identified for impaired R1 (red, purple and grey nodes (Fig. 3.1(A)) and unimpaired R1 (green, blue and purple nodes (Fig. 3.2(A), however the muscle they included did not necessarily match across groups. FCU and ADM were affiliated with APB and ED among unimpaired controls but for post-stroke participants instead merged into the larger red submodule (including DC, TB, BR and BB), leaving APB and ED as a smaller functional submodule among impaired participants (purple nodes). Within the unimpaired R2 and impaired R4 network pairing, three submodules were also found in each but the same pattern of specific muscles (BR and ED) merging into larger submodules persisted. Moreover, this same pattern of merging was displayed in the impaired R3 unimpaired R3 and R4 pairings. The light green submodule of unimpaired R3 was reflected in the light green submodule of impaired R3 which also reflected the grey submodule of unimpaired R4 (orange submodule (R3 Fig. 3.2(A)), suggesting a merging of unimpaired motor patterns. In contrast, for the R5 network pairing between groups, the unimpaired group displayed larger submodules than the post-stroke sample (i.e. light green nodes of unimpaired R5 include 7 muscles while the largest submodule in the impaired R5 (dark green nodes) included just 5 muscles). This largest submodule of the unimpaired group (light green nodes) comprised of muscles across the upper- and lower-arm while the post-strokes’ largest submodule (dark green) was exclusive to muscle from the elbow up. Together, these observations suggest both merging and fractionation of functionally similar modules was present among the local network interactions of post-stroke participants.

Finally, to characterise these submodular structural differences in functional connectivity between groups, we applied the NBS to the multiplex network with network layers corresponding to participant- and kinematic-specific interactions. We found no redundant muscle interactions were consistently greater across kinematics for the unimpaired controls compared to post-stroke (Fig. 3.4(A)), while several redundant muscle interactions from the post-stroke sample were significantly greater (the magnitude of this difference represented by the edge-width) than their unimpaired counterparts (Fig. 3.4(B)). These significantly greater magnitude interactions included hand, elbow and shoulder level muscles namely: APB-ECU, BR-FCR, BB-FCU, DC-FCU and DC-ECU.

**Fig. 3:**
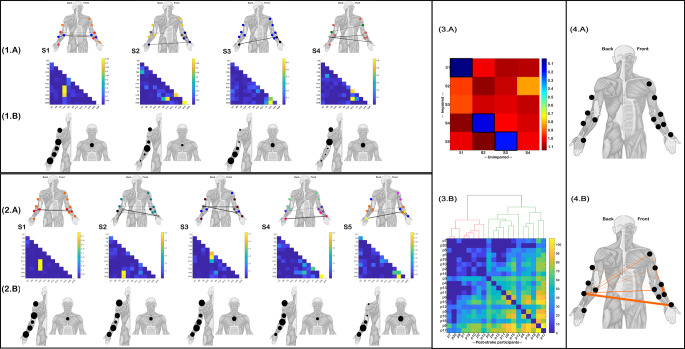
The functionally-similar muscle networks extracted from unimpaired controls **(1.A)** and stroke survivors (**2.A**) accompanied by human body models illustrating their most prominent functional connectivities and their relative magnitudes (edge-width) and submodular structure (node colour). The corresponding kinematic-specific activation coefficients averaged across participants are illustrated as the relative size of nodes on human body models below their unimpaired **(1.B)** and impaired (**2.B**) networks. (**3.A**) A representational-dissimilarity matrix (RDM) illustrating the distance (i.e. 1-r) between pairs of impaired (rows) and unimpaired (columns) muscle networks. The most closely matched networks are indicated by the black framed squares. (**3.B**) The aggregated RDM derived from computing the distance (i.e. 1-r) between each pair of impaired and unimpaired participants’ kinematic-specific muscle network and then performing a one-mode projection for each RDM to define post-stroke participant distance matrices. The illustrated RDM is the summation of these distance matrices across kinematics, illustrating the overall distance of each stroke survivor (p1-p20) from the unimpaired controls. Above this aggregated RDM, the output from an agglomerative clustering across the set of kinematic-specific distance matrices illustrates the clustering of the corresponding participants into red and green clusters. (**4.A**) Between-group comparison of the multiplex networks of functionally similar muscle interactions for each participant and kinematic using NBS revealed no significantly greater interaction magnitudes for the unimpaired cohort but, as shown in (**4.B**), significantly greater interaction magnitudes were found among the post-stroke sample. Edge-widths indicate the cluster-level differences between groups for the presented interactions

### Functionally-complementary muscle networks

Four synergistic muscle networks were identified and extracted from the unimpaired controls (Fig. 4.1(A)) while 5 synergistic muscle networks were found among the post-stroke sample (Fig. 4.2(A)). Unimpaired S1-S3 appeared to be conserved in the post-strokes’ S1, S4 and S5 respectively with a high degree of similarity observed (i.e. 1-*r* < 0.2 (Fig. 4.3(A))). S1 for both groups, mirroring R1 previously (see Fig. 3.1–2(A)), was comprised of prominent couplings between FDS and both PT and EDU (Fig. 4.1–2(A)). S2 had more widespread couplings, between ADM-APB, FCU-APB, PT-FCR and to a lesser extent BR-APB and DC-FDS. For S3, the most prominent couplings were found among FCR and ADM and to a lesser extent FCR and APB. The kinematic activation of these three structurally conserved muscle networks was similar for S1, with equivalent scaling’s for all kinematics found in both groups. For unimpaired S2 and impaired S4, the pattern of kinematic activation was also similar however there were fewer differences between the more highly and lowly scaled kinematics among post-stroke participants, suggesting greater inter-participant variability in the mapping of muscle interactions to task performance among stroke survivors. Further, the kinematic scaling of muscle interactions in unimpaired S3 (and impaired S5) was also similar, with the forearm scaling most prominently in both groups. Turning to the muscle networks unique to the post-stroke group (i.e. S2 and S3 (Fig. 4.2(A))), S2 was comprised of prominent couplings between FDS and both ADM and APB. These muscle couplings only featured in a minor way among the unimpaired group in S1 (Fig. 4.1(A)). Impaired S2 scaled prominently for all kinematic markers except the elbow and chest, matching the kinematic activation of impaired and unimpaired S1 (Fig. 4.1–2(B)). Impaired S3 comprised of strong functionally complementary interactions between ED and FCU, BR and to a lesser extent ECU. Again, unimpaired S1 contains the only functional connections that could reflect this post-stroke coordination pattern with subtle couplings among these muscles. S4 of the unimpaired controls represented a muscle network specific to this group and was composed of prominent functional connectivity between ECU and both APB and PT. These functional connections were present at a very low relative magnitude in the impaired S2 and S4 (as evidenced also in their representational similarity (Fig. 4.3(A)).

Probing the structural alteration of synergistic muscle networks in chronic stroke survivors at a global level, the agglomerative clustering of RSA matrices from matching individual unimpaired and impaired participants across kinematics revealed two patient subgroups (red and green clusters (Fig. 4.3(B)) distinct from those identified among redundant muscle networks (Fig. 3.3(B)). Here, the green cluster consisted of 12/20 less impaired stroke survivors with significantly greater FMA-UE scores (18.5 ± 1.7 vs. 16.6 ± 2.1, (t= -2.2 (*p* < 0.05))) and MMSE scores (28.75 ± 0.97 vs. 26.62 ± 1.19, (t= -4.2 (*p* < 0.001))) compared to the red cluster members. These differences in cognitive and functional performance between subgroups were underpinned (as found amongst the redundant network clustering’s (Fig. 3.3(B))) by a greater total distance of the less impaired green cluster from the control group in terms of their muscle network structure (Fig. 4.3(B)). This insight again demonstrates that the neurological deficits following stroke are more effectively compensated for among less impaired individuals by adopting novel motor patterns, an insight that is prominently reflected amongst the synergistic muscle interactions quantified here.

Despite the structural similarity of S1 of both groups (Fig. 4.1–2(A)), their submodular organisation was noticeably different. More specifically, the post-stroke cohort displayed two submodules in S1 (orange and red nodes), whereas the unimpaired controls had three (orange, red and blue nodes). The main difference between groups for this network component was that the orange submodule including the upper arm muscles in both groups was more widespread in the post-stroke sample, additionally including ADM, FCU, BR and ED. In contrast, for the unimpaired S2 – impaired S4 pairing (Fig. 4.1–2(A)), the latter displayed 4 submodules compared to just 3 in the unimpaired cohorts S2. Moreover, the unimpaired S3 – impaired S5 combination further illustrated this difference in submodular structure, with 2 vs. 4 submodules identified respectively. More prominent couplings between the APB and PT and between DC and BB among the post-stroke sample appear to underly this difference in functional organisation. To comprehensively characterise these alterations in local network sub-clustering in chronic stroke, the application of NBS revealed significantly greater interaction magnitudes among the post-stroke sample compared to the controls across kinematics (orange network edges (Fig. 4.4(B))). No synergistic muscle interactions were typically greater in magnitude among the unimpaired controls on the other hand (Fig. 4.4(A)). Interactions between upper- and lower-arm musculature (e.g. DC-FDS, DC-APB and TB-PT) but also between both upper- and forearm muscles with hand musculature (e.g. ECU-APB, DC-APB) were common among these significantly greater functional connectivities. The most prominent differences (as indicated by edge-width) were observed for ECU-APB and BR-FCR.

**Fig. 4:**
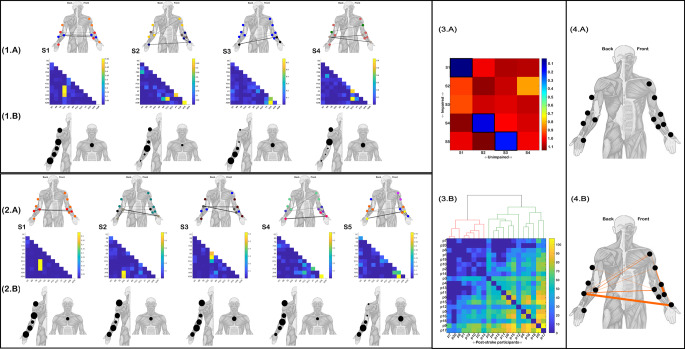
The functionally-complementary muscle networks extracted from unimpaired controls (**1.A**) and stroke survivors (**2.A**) accompanied by human body models illustrating their most prominent functional connectivities and their relative magnitudes (edge-width) and submodular structure (node colour). The corresponding kinematic-specific activation coefficients averaged across participants are illustrated as the relative size of nodes on human body models below their unimpaired (**1.B**) and impaired (**2.B**) networks. (**3.A**) A representational-dissimilarity matrix (RDM) illustrating the distance (i.e. 1-r) between pairs of impaired (rows) and unimpaired (columns) muscle networks. The most closely matched networks are indicated by the black framed squares. (**3.B**) The aggregated RDM derived from computing the distance (i.e. 1-r) between each pair of impaired and unimpaired participants’ kinematic-specific muscle network and then performing a one-mode projection for each RDM to define post-stroke participant distance matrices. The illustrated RDM is the summation of these distance matrices across kinematics, illustrating the overall distance of each stroke survivor (p1-p20) from the unimpaired controls. Above this aggregated RDM, the output from an agglomerative clustering across the set of kinematic-specific distance matrices illustrates the clustering of the corresponding participants into red and green clusters. (**4.A**) Between-group comparison of the multiplex networks of functionally complementary muscle interactions for each participant and kinematic using NBS revealed no significantly greater interaction magnitudes for the unimpaired cohort but, as shown in (**4.B**), significantly greater interaction magnitudes were found among the post-stroke sample. Edge-widths indicate the cluster-level group differences for the presented interactions

### Functionally-independent muscle networks

Five functionally-independent modules were identified and extracted from both unimpaired and impaired groups (U1-U5 (Fig. 4.1–2(A))). These modules are presented as bar graphs illustrating the average unique information for each muscle when paired with every other muscle as they do not represent functional muscle couplings. To the left of each bar graph, the average unique information values are presented as proportionately scaled nodes on a human body model while their corresponding kinematic-specific activations are presented below. The structure of unimpaired U1- U4 most closely corresponded to U1, U4, U5 and U3 respectively (black framed squares (Fig. 4.3(A))). More specifically, in both groups U1 was comprised of high unique information on average for FDS followed by lower magnitude independence among ECU, PT and FCR. The scaling of U1 was relatively uniform across kinematics in both groups. U2 of the post-stroke sample had an obvious correspondence with the impaired U1 and unimpaired U1 with prominent functional independence for FDS. Nonetheless, a key difference for this module was the greater functional independence among muscles of the hand (i.e. ADM, APB) and lower functional independence among other forearm muscles (i.e. PT, ECU), resulting in relatively low structural similarity (Fig. 4.3(A)). There were also no clear differences in the kinematic scaling of impaired U2 compared to impaired U1. For the unimpaired U2 and impaired U4 combination, APB had the highest functional independence typically from all other muscles. In contrast to unimpaired U2 however, impaired U4 had a noticeably greater involvement of ECU and slightly greater relative independence among several other muscles. Moreover, the kinematic scaling for this pair of networks differed to some extent, with the post-stroke sample demonstrating greater involvement of the elbow while the forearm was most prominently scaled for the controls. The more uniform scaling of kinematics among the post-stroke sample was again observed here as a key difference with the more distinguished kinematic activation of unimpaired U2. Turning to unimpaired U3 which corresponded most closely with impaired U5, FCR was typically most prominent in its functional independence from all other muscles, followed by ADM and APB. Impaired U5 had noticeably greater functional independence for ED, BR, ECU, BB and DC while its kinematic scaling also differed with greater involvement of the chest and shoulder kinematic. Finally for unimpaired U4 and the corresponding impaired U3, both were comprised of high functional independence for ED. The unimpaired sample demonstrated greater relative functional independence for DC in this module pairing but less so for BR. The kinematic scaling of this module pairing was mostly consistent, however again, the unimpaired activations were more proportionately distinguishable compared to the post-stroke sample.

Clustering the RDMs derived from matching impaired and unimpaired participants’ functionally independent network structures across each kinematic identified two subgroups of post-stroke participants (red (12 participants) and green (8 participants) clusters (Fig. 5.3(B))) that were distinct from those identified from redundant and synergistic clusterings (see Figs. 3-4.3(B)). The subgroupings identified here significantly differentiated participants based on MMSE scores (red subgroup: 27.33 ± 1.5, green subgroup: 28.75 ± 1.04; t= -2.5 (*p* < 0.05)) but not for FMA-UE scores (red subgroup: 17.4 ± 2.1, green subgroup: 18.25 ± 1.98; t= -8.98 (*p* > 0.05)), suggesting cognitive impairment had an influential role in the structural alterations of functionally independent muscle interactions post-stroke. As with the coarse-grained control mechanisms reflected in the synergistic and redundant muscle networks (see Figs. 3-4.3(B)), the recruitment of novel patterns of individual muscle control here was also associated with the less impaired subgroup (greater total distance values in the aggregated RDM of the green cluster (Fig. 5.3(B))).

Finally, we identified several muscle pairs that had significantly different magnitudes of unique information between groups, differences which were present for both groups (unimpaired group (Fig. 5.4(A)) and impaired group (Fig. 5.4(B))). Magenta network edges indicate greater functional independence of a muscle located more proximally than the paired muscle and vice versa for cyan edges. The significantly different interactions in favour of the unimpaired group (Fig. 5.4(A)) were more subtle than those favouring the post-stroke sample (Fig. 5.4(B)), the greatest difference (as indicated by network edge-width) being the task information uniquely provided by APB relative to ECU (Fig. 5.4(B)). This was followed by considerable differences in favour of the post-stroke sample for FCR relative to BR and TB relative to PT. Meanwhile, the most consistently different functionally independent interaction across kinematics in favour of the unimpaired cohort was FDS relative to FCU. Overall, these findings suggest alterations in the independent control of muscles in chronic stroke survivors are complex, with functional independence being either exacerbated or reduced depending on the muscle combination.

**Fig. 5:**
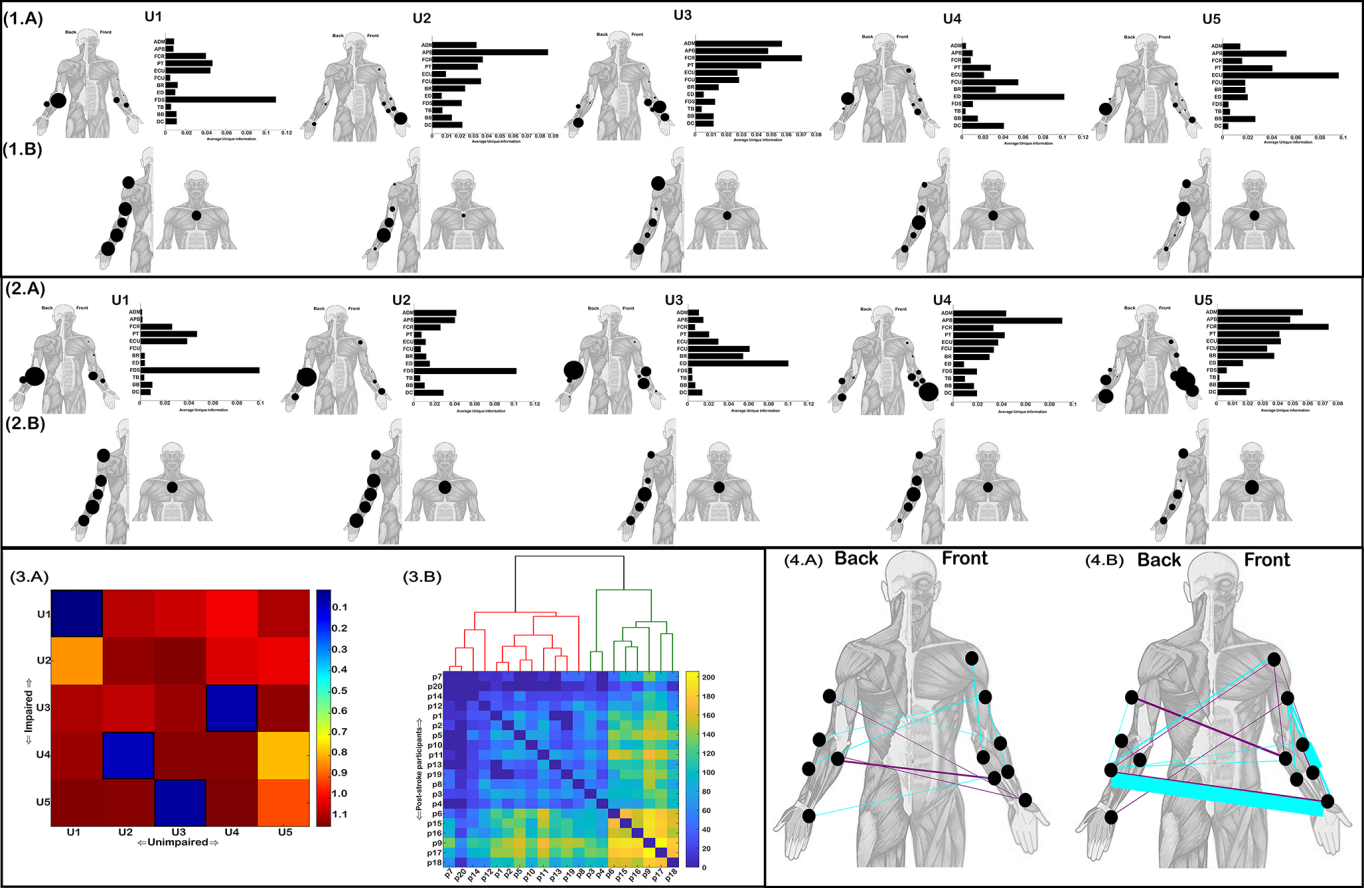
The functionally-independent muscle modules extracted from unimpaired controls (**1.A**) and stroke survivors (**2.A**) illustrated as bar graphs with the height of bars indicating the average unique information of each muscle from all other muscles. Each bar graph is accompanied by human body models further illustrating the magnitude of these average unique information values (proportional node size). The corresponding kinematic-specific activation coefficients averaged across participants are illustrated as the relative size of nodes on human body models below their unimpaired (**1.B**) and impaired (**2.B**) modules. (**3.A**) A representational-dissimilarity matrix (RDM) illustrating the distance (i.e. 1-r) between pairs of impaired (rows) and unimpaired (columns) muscle components. The most closely matched components are indicated by the black framed squares. (**3.B**) The aggregated RDM derived from computing the distance (i.e. 1-r) between each pair of impaired and unimpaired participants’ kinematic-specific muscle component and then performing a one-mode projection for each RDM to define post-stroke participant distance matrices. The illustrated RDM is the summation of these distance matrices across kinematics, illustrating the overall distance of each stroke survivor (p1-p20) from the unimpaired controls. Above this aggregated RDM, the output from an agglomerative clustering across the set of kinematic-specific distance matrices illustrates the clustering of the corresponding participants into red and green clusters. (**4.A**) Between-group comparison of the multiplex network of functionally independent muscle interactions across kinematics using NBS revealed significantly greater interaction magnitudes for the unimpaired and impaired (**4.B**). Magenta network edges indicate unique information from a more proximal muscle relative to a more distal muscle and vice versa for cyan edges. Edge-widths indicate the cluster-level group differences for the presented interactions

## Discussion

The aim of this study was to address shortcomings in current clinical assessment tools for chronic stroke survivors by characterising the alteration of functional muscle interactions post-stroke through a novel perspective to the muscle synergy. Towards this aim, we applied our newly established computational framework for the first time to 20 chronically impaired stroke survivors and 17 unimpaired participants, where participants each performed single-trials of a forward pointing movement. This application unveiled networks of functionally diverse types of muscle interaction that were both shared across and specific to each experimental group, along with salient groups differences in the submodular structure of networks from each interaction type (i.e. redundant, synergistic and unique). Furthermore, based on these structural differences, we identified novel patient subgroupings that were associated with established measures of functional and/or cognitive impairment. These subgroupings distinguished less impaired stroke survivors that adopted novel motor patterns distinct from unimpaired controls from more severely impaired patients that did not recruit novel motor patterns. Our findings shed new light on motor impairment post-stroke by characterising the impairment of diverse types of muscle interaction (i.e. functionally-similar, -complementary and -independent).

### New and congruent insights into the characteristic alterations of motor function in chronic stroke survivors

In several ways our findings aligned with previous muscle synergy research on the post-stroke population. Among these congruent findings, we found that the number and structure of extracted muscle networks was mostly preserved between healthy controls and chronic stroke survivors (Cheung et al. 2009; Roh et al. 2013), with the exception of synergistic muscle networks (impaired group = 5 vs. unimpaired group = 4). Functionally-similar (i.e. redundant) and -independent (i.e. unique) muscular dependencies are, conceivably, quantifiable using current muscle synergy analysis as shared and muscle-specific patterns of activation respectively, however, synergistic interactions are unique to our framework employed here. Thus, the application of our framework went beyond established findings, providing a novel insight in the greater number of synergistic modules employed in post-stroke movements. Continuing, within each interaction type (i.e. redundant, synergistic, unique), we found most networks’ corresponded well across groups with the exception of 1–2 components from either group. Nevertheless, several of these functional modules shared across groups demonstrated key differences in their submodular organisation indicative of re-weighting dependencies, merging and fractionation (Cheung et al. 2012). Moreover, several of the group-specific modules corresponded qualitatively also, to different extents, with merged or fractionated versions of modules from the other groups. These concurrent phenomena can be summarised as three different kinds of impairment response: (a) the preservation of healthy functional modules, (b) the systematic reweighting, merging and fractionation of muscle interactions resulting in structural changes to modularity at various scales, and (c) the adoption of new and/or loss of existing functional modules (Cheung et al. 2009, 2012; Cirstea and Levin 2000; Clark et al. 2010; Houston et al. 2021; Roh et al. 2013). The co-occurrence of these different impairment responses at the group level among chronic stroke survivors reconciles results from previous studies that observed these patterns separately (Cheung et al. 2009; McMorland et al. 2015; Roh et al. 2013), showing that they are all present at the group level and indeed persist across different types of muscle interaction.

### A whole-limb perspective to post-stroke motor impairment

The analysis of a simple forward pointing movement by the affected upper-limb here brought about interesting insights, in particular the NBS results highlighting differences in submodular composition (i.e. clusters of altered muscle interaction weightings) (see Figs. 3–5.4(A-B). More specifically, for this shoulder-led movement, consistent group differences across kinematics were found across all interaction types predominantly for interactions among finger flexor/extensors and with the hand muscles (e.g. BR-FCR, ECU-APB). Although some significant differences were found involving the shoulder muscle (DC), these differences were much weaker than those among distal muscles. This finding contrasts with established work suggesting shoulder-related muscle synergies are a focal point of abnormal function among chronic stroke survivors during an isometric, shoulder-led task (Roh et al. 2013). Instead, our finding aligns with more recent work with an isometric upper-limb task showing that abnormal couplings were strongest among paretic wrist/finger muscles during proximal muscle contractions and weakest when the coupling was elicited by distal musculature (McPherson and Dewald 2022). Indeed, it was specifically noted in (McPherson and Dewald 2022) that this pattern was most prominent for extrinsic wrist extensors and intrinsic hand muscles, as specifically found here with the interaction between ECU (an extrinsic wrist extensor) and APB (an intrinsic hand muscle) being consistently most different between groups for redundant, synergistic and unique modules. This discrepancy with previous work may be explained by novel features of our framework employed here, such as the move-away from optimising variance accounted for (VAF) metrics which may emphasize higher amplitude muscles (e.g. the deltoids) at the expense of more subtle but functionally relevant muscle couplings (e.g. hand and wrist extensor muscles) (Alessandro et al. 2013). Although abnormal function at the shoulder level is undoubtably present among chronic stroke survivors (Brunnstrom 1970; Houston et al. 2021; Roh et al. 2013), our findings shed new light on the complex interactions underlying impaired arm movements, showing that they manifest in different ways across the whole limb. Further, we have also shed light on how they may manifest, for example in the stronger weighting of specific muscle couplings (i.e. Figs. 3–4.4) and in the complex interchange of selective muscle control (see Fig. 5.4), both proximally and distally.

### Stroke survivors broadly cluster into clinically relevant patient subgroups

Finally, through RSA we identified a global bipartition of chronic stroke survivors among each interaction type (i.e. redundant, synergistic and unique) that each included patients that shared relatively similar structural differences with unimpaired controls. The redundant and unique derivations of these subgroupings discriminated participants based on cognitive impairment only while synergistic networks were sensitive to both cognitive and functional impairments. Although redundant, synergistic and unique patient partitions were all composed of a majority group, they did not cluster participants in the same way, with different participants affiliated with different groups in each interaction type (see Figs. 3–5.3(B). Moreover, the majority cluster from the synergistic partition represented the less impaired subgroup while the majority clusters for redundant and unique partitions represented the more severely impaired. This suggests that the different interaction types quantified here capture different contributory factors underlying post-stroke impairment. Moreover, and perhaps counterintuitively when considering previous studies (Funato et al. 2022; Hong et al. 2021; Liu et al. 2024; Scotto di Luzio et al. 2022; Zhao et al. 2023, 2024), the less impaired subgroup was consistently more different from the unimpaired participants compared to the more severely impaired cohort across all three interaction types. This observation points towards the superior recovery of some stroke survivors who, instead of recovering healthy motor patterns as commonly thought (Cirstea and Levin 2000), adopted novel motor patterns as shown in other research (García-Cossio et al. 2014).

It also highlights that a common clinical practice in using ‘*similarity to healthy controls’* metrics to assess motor function in less impaired clinical populations may be misleading, at least through the lens of our framework. The patient clustering’s we observed instead suggest that exploiting the available degrees-of-freedom of less impaired patients by promoting the exploration of the task space and therefore the recruitment of adaptive compensatory strategies and novel motor patterns (Jones 2017), although still atypical, may be beneficial towards their overall motor function. This distinct interpretation provided by our framework aligns with previous work demonstrating a two-way stratification of stroke survivors into those whose recovery was and was not proportional to their initial impairment (Bonkhoff et al. 2022; Prabhakaran et al. 2008), representing distinct recovery patterns linked to sensorimotor cortex integrity (Byblow et al. 2015; García-Cossio et al. 2014; Young et al. 2022). Our findings here concur with this observation, demonstrating that less cognitively impaired patients were able to adopt novel motor patterns likely through the adaptation of alternative descending neural pathways (Jones 2017; McMorland et al. 2015), while more severely impaired cohorts were not, together suggesting direct links with neural impairment post-stroke. Future work should further investigate these patient subclusters and the distinct types of muscle interaction that underpin them both physiologically in terms of their underlying neural substrates and functionally in terms of key indicators of their behavioural manifestation (Alessandro et al. 2013; Cheung and Seki 2021; Delis et al. 2010).

## Limitations

The presented findings are limited to the motor task analysed in this study which did not explore the full range of arm movements. The clustering approach employed here defined a global partition of stroke survivors into two subgroups each of which, as found in previous work (Scano et al. 2017), were composed of more refined patient subclusters that may represent important functional and physiological differences in the stroke population (see Figs. 3–5.3(B). These subclusters have not been explored using the NIF and remain a topic of future research. Finally, the neurophysiological underpinnings for the different types of functional muscle interaction are currently unknown. In future work we aim to incorporate brain and spinal level neural signals into the NIF to provide a holistic perspective to the neurocomputational principles of human motor behaviour and characterise the neural bases of motor impairment.

## Conclusion

From single trials of a simple forward pointing movement, the current study presents novel insights into the motor impairment of chronic stroke survivors by implementing a recently proposed computational framework to extract networks of muscle interactions with diverse functional consequences. Specifically, we have shown that chronic stroke survivors can be broadly categorised into two clinically meaningful subgroups depending on the extent of preservation of healthy motor patterns. Through group-level statistical tests, we have also shown that subtle, abnormal muscle interactions among distal musculature due to proximal muscle contractions are persistent across the post-stroke cohort and involve different types of muscle interaction, promoting a whole-limb perspective to post-stroke impairment. Finally, our findings both aligned with and built significantly on previous research by adding substantial nuance to the analysis of muscle synergies in this clinical population (Cheung et al. 2009; Cirstea and Levin 2000; Clark et al. 2010; Houston et al. 2021; Roh et al. 2013), characterising how functionally-similar, -complementary and -independent muscle interactions are typically altered post-stroke. In summary, our work here paves new avenues towards progressing our understanding of post-stroke motor impairments and the clinical use cases for muscle synergy analysis.

## Data Availability

This study conducted a secondary analysis of opensource data (‘MHH’) found here (Averta et al. 2021). To support the further progression of the research presented here, we have uploaded the patient demographic data, extracted muscle networks and subgroupings at the following repository: https://figshare.com/articles/dataset/26303404. Matlab code for the framework can be found here: https://github.com/DelisLab/Muscle_PID.
